# Association of sleep duration with metabolic syndrome and its components in children and adolescents; a propensity score-matched analysis: the CASPIAN-V study

**DOI:** 10.1186/s13098-018-0381-y

**Published:** 2018-11-03

**Authors:** Zeinab Hemati, Nafiseh Mozafarian, Ramin Heshmat, Zeinab Ahadi, Mohammad Esmaeil Motlagh, Hasan Ziaodini, Majzoubeh Taheri, Tahereh Aminaee, Mostafa Qorbani, Roya Kelishadi

**Affiliations:** 1 Child Growth and Development Research Center, Research Institute for Primordial Prevention of Non-communicable Disease, Isfahan University of Medical Sciences, Hezar-Jarib Ave, Isfahan, Iran; 2 Chronic Diseases Research Center, Endocrinology and Metabolism Population Sciences Institute, Tehran University of Medical Sciences, Tehran, Iran; 3 Department of Pediatrics, Ahvaz Jundishapur University of Medical Sciences, Ahvaz, Iran; 4 Office of Health and Fitness, Ministry of Education, Tehran, Iran; 5 Bureau of Population, Family and School Health, Ministry of Health and Medical Education, Tehran, Iran; 6 Non-communicable Diseases Research Center, Alborz University of Medical Sciences, Karaj, Iran; 7 Endocrinology and Metabolism Research Center, Endocrinology and Metabolism Clinical Sciences Institute, Tehran University of Medical Sciences, Tehran, Iran

**Keywords:** Metabolic syndrome, Cardiovascular risk factors, Sleep duration, Children, Adolescents

## Abstract

**Objective:**

This study aims to evaluate the association of sleep duration with metabolic syndrome (MetS) and its components in a pediatric population.

**Methods:**

This multi-centric cross-sectional study was conducted in 2015 in 30 provinces of Iran. Participants consisted of 4200 school students aged 7–18 years, studied in a national school-based surveillance program (CASPIAN-V). Physical examinations and laboratory tests were performed using standard protocols. The analysis was conducted based on the propensity score matching and conditional logistic regression was used to evaluate the association of short sleep (less than 8 h a day) and the onset of sleep with MetS and its components. Results of conditional logistic regression was reported as odds ratios (OR) and 95% confidence intervals (CI).

**Results:**

Overall, 3843 of participants completed the survey (response rate: 91.5%). Their mean (SD) age was 12.3 (3.2) years and 50.6% were boys. In the multivariate model, individuals who slept less than 8 h a day had significantly higher odds of MetS (OR 2.05, 95% CI 1.19–3.63) and high blood pressure (BP) (OR 1.46, 95% CI 1.04–2.06). Association between short sleep duration with other MetS components (including abdominal obesity, hypertriglyceridemia, hyperglycemia, and low levels of high-density lipoprotein-cholesterol was not statistically significant (P > 0.05). Moreover, association between the onset of sleep with MetS and its components was not statistically significant (P > 0.05).

**Conclusion:**

Short sleep duration is associated with increased risk of MetS and high BP in children and adolescents. The clinical impact of current findings should be assessed in future longitudinal studies.

## Introduction

Chronic diseases have emerged as a rapidly increasing public health problem in developing countries [1, 2]. Interest in childhood precursors to chronic diseases, particularly cardiovascular disease (CVD), is increasing because both lifestyle and biological risk factors of such diseases persist from childhood into adulthood, and the several cardiometabolic risk factors, including diabetes, obesity, dyslipidemia and metabolic syndrome (MetS), are followed from childhood to adult life and diseases [1, 3, 4].

Some lifestyle variables, including physical inactivity, unhealthy dietary habits, and insufficient sleep are associated with the development of CVD in later life [5, 6]. Due to multiple factors, most adolescents do not obtain sufficient sleep [7]. Previous studies have reported that average daily sleep duration in children and adolescents has declined by ~ 70 min in the past century [8].

Short sleep duration in children and adolescents is associated with increased BMI [9–13]. One study found that children ages 3.6–18.5 years with less sleep duration have more insulin resistance than children with normal sleep times [14]. Another study in Hong Kong reported short sleep duration increased hypercholesterolemia in a longitudinal study of adolescents [15]. In a recent study on 238 adolescents age 13–16 years, the odds of prehypertension was increased 2.5-fold for short sleep [16]. Other studies have shown a significant association between short sleep duration and cardiometabolic risk factors in children, such as dyslipidemia, and glucose homeostasis [17–20]. As a mechanism, a study showed that short sleep duration was associated with elevated ghrelin levels and reduced leptin, which are known to control body weight. In a randomized crossover clinical trial, sleep deprivation was associated to elevations in the orexigenic factor ghrelin, reductions in the anorexic hormone leptin, and increased hunger and appetite [21, 22].

Similar to many other developing countries, the epidemiologic transition along with rapid lifestyle changes has made Iranian children prone to MetS, and, as a result, to chronic diseases morbidity and mortality in adulthood [17, 23, 24]. Therefore, this study was conducted to examine the association of sleep duration with MetS and its components in a large nationally-representative sample of children and adolescents.

## Methods

The present study was part of “the fifth survey of the school-based surveillance system entitled Childhood and Adolescence Surveillance and Prevention of Adult Non-communicable Disease (CASPIAN-V) study” (2014–2015) conducted in 30 provinces of Iran. Details on the study protocol have been discussed previously [25], and here we briefly point to the main parts.

### Study population and sampling

Using multistage stratified cluster sampling method, the study participants were selected from 7 to 18-year-old school students living in urban and rural areas of 30 provinces.

For proportional to size sampling along with the student’s residence area (urban/rural), educational levels (primary/secondary) were considered with equal sex ratio [26].

Achieving the desired number of samples was obtained using cluster sampling in each province with equal cluster sizes. Clusters were determined at school levels. The size of each cluster was 10 students; meaning that a total of 10 statistical units (including 10 students and their parents) would be considered in each cluster. The sample size of main survey included 480 students in each province (48 clusters of 10 students), i.e. a total of 14,400 students at national level. In each province, 14 out of 48 clusters were randomly selected for biochemical tests. Therefore, sample size of current study was estimated to be 4200.

#### Procedure and measurements

Data were collected by the Persian version of the World Health Organization-Global School Student Health Survey (WHO-GSHS) and its validity and reliability have been assessed previously [27]. Two sets of questionnaires were gathered from students and their parents.

Students’ demographic characteristics (including age, sex and….), healthy behaviors (physical activity, screen time and sleep duration) and dietary habit were asked through students’ questionnaires and some complementary data on family characteristics, namely household size, order of students and socioeconomic variables questioned through parents’ questionnaires.

Physical examination was performed under standard anthropometric techniques by a team of trained health care experts. Weight was measured to the nearest 0.1 kg using a calibrated scale placed on a flat ground and height was measured to the nearest 0.1 cm using a portable stadiometer [28].

Waist circumference (WC) was measured to the nearest 0.1 cm 3 times and the average of three values was used for the analyses. A non-elastic tape was used to measure WC at a point midway between the lower border of the rib cage and the iliac crest at the end of normal expiration [29]. A mercury sphygmomanometer was used to measure blood pressure (BP) on the right arm while participants were in the sitting position. BP was measured 2 times at 5 min intervals, and the average of the two values was used for the analyses [30].

After 12 h overnight fasting, venous blood sample was collected from students. Fasting blood glucose, triglycerides (TGs), and high density lipoprotein cholesterol (HDL-C) were measured enzymatically by Hitachi Auto Analyzer (Tokyo, Japan) [31, 32].

#### Definition of terms

To evaluate the screen time (ST) behaviors, using the WHO-GSHS questionnaire, the students were asked to report the numbers of hours per day that spent watching television (TV) and/or videos, personal computer, or electronic games on week days and at weekends. Then, the total cumulative time spent on these activities was calculated and ST was categorized into two groups; less than 2 h/day was defined as low, and 2 h/day or more was defined as high group [33].

Students were asked to report the average number of hours/day spent for sleeping at weekdays and weekends. According to previous studies, short sleep duration was defined as sleep duration ≤ 8 h/day [12, 17]. Students were also asked to report the onset of sleep occasionally.

Socioeconomic status (SES) included father’s job and education, mother’s job and education, having private car and computer, and type of student’s school (private, public) variables that were summarized in one main component, by principle component analysis (PCA) method. SES was categorized into tertiles. The first tertile was defined as a low SES, second tertile as a moderate and third tertile as a high.

To define healthy and unhealthy eating behaviors, students were asked to determine frequency of consumption of breakfast, fruit, vegetables, milk, sugar sweetened beverages (carbonated drinks and synthetic fruit juices), fast foods (sausages, hamburgers and pizza), sweets (cakes, candies, biscuits and chocolates) and salty snacks (chips, pretzels and salty puff pates). According to PCA method, two factors were loaded in PCA method; in the first factor, consumption of breakfast, fruit, vegetables and milk were loaded which defined as healthy eating behaviors and in the second factor intake of sugar sweetened beverages, fast foods, sweets and salty snacks were loaded which defined as unhealthy eating behaviors. Healthy and unhealthy eating behaviors factor was categorized into tertiles. The first tertile was defined as a low, second tertile as a moderate and third tertile as a high.

Physical activity (PA) was assessed through the validated questionnaire [34]. Using self-administrated questionnaire students were asked about 7-day recalls of sports or activities in and out of school activities that led to heavy sweating or significant increase in heart rate, or games that make students breathe hard. All physical activity items were summarized in a main variable using principal component analysis (PCA) methods and then this main variable was categorized into tertiles. The first tertile was defined as a low, second tertile as a medium and third tertile as a high PA [35].

According to definition of Cook et al. (National Cholesterol Education Program Expert Panel on Detection, Evaluation and Treatment of High Blood Cholesterol in Adult Treatment Panel III) participants were recognized as having MetS if they had three out of the following criteria: fasting TG ≥ 110 mg/dl; HDL-C ≤ 40 mg/dl; WC ≥ 90th percentile for age and sex, according to national reference curves; systolic BP (SBP) and/or diastolic BP (DBP) > 90th percentile for sex, age and height, from national reference cut-off points; and FBG ≥ 100 mg/dl [36, 37].

#### Ethical concerns

Approval for the study was obtained from the relevant local and the national committees (The Research and Ethics council of Tehran and Isfahan University of Medical Sciences, Project number: 194049).

#### Statistical analysis and matching based on Propensity Score (PS)

Using STATA package ver. 11.0 (Stata Statistical Software: Release 11. College Station, TX: Stata Corp LP. Package). Results provide as mean and standard deviation (SD) for continuous variables, and as number (percentage) for categorical variables.

Comparison of percentages of the categorized variables was made using the Pearson Chi square test. PS was calculated based on conditional logistic model with potential confounding variables (age, sex, living area, SES, PA, ST, healthy and unhealthy eating behaviors, family history of chronic diseases and parental BMI). Two groups (with MetS and without MetS) were matched based on 1:1 matching method without replacement by the score. Then standardized skewness (SS) percentages before and after synchronization were calculated and then SS mean was calculated for all variables. Due to PS matching, conditional logistic regression was used to evaluate the association of the short sleep duration (as categorical variable) and the onset of sleep (as continuous variable) with MetS and its components. Results of conditional logistic regression was reported as odds ratios (OR) and 95% confidence intervals (CI). All statistical analysis were estimated using survey data analysis methods. P < 0.05 was considered as statistically significant.

## Results

Overall, 3843 students (52.3% boys) with mean (SD) age of 12.45 (3.04) years from 4200 invited students (participation rate: 91.5%) were participated in the current study. The mean (SD) duration of sleep was 8.57 (1.23) and the frequency of short sleep was 33.5% (sleep duration ≤ 8 h/day). The prevalence of MetS was 5% (4.5% in girls and 5.5% in boys, P-value: 0.17) and the most common MetS components was low HDL-C (29.5%). The frequency of 0, 1, 2 components of Mets was 38.7, 36.4 and 19.9% respectively.

**Fig. 1:**
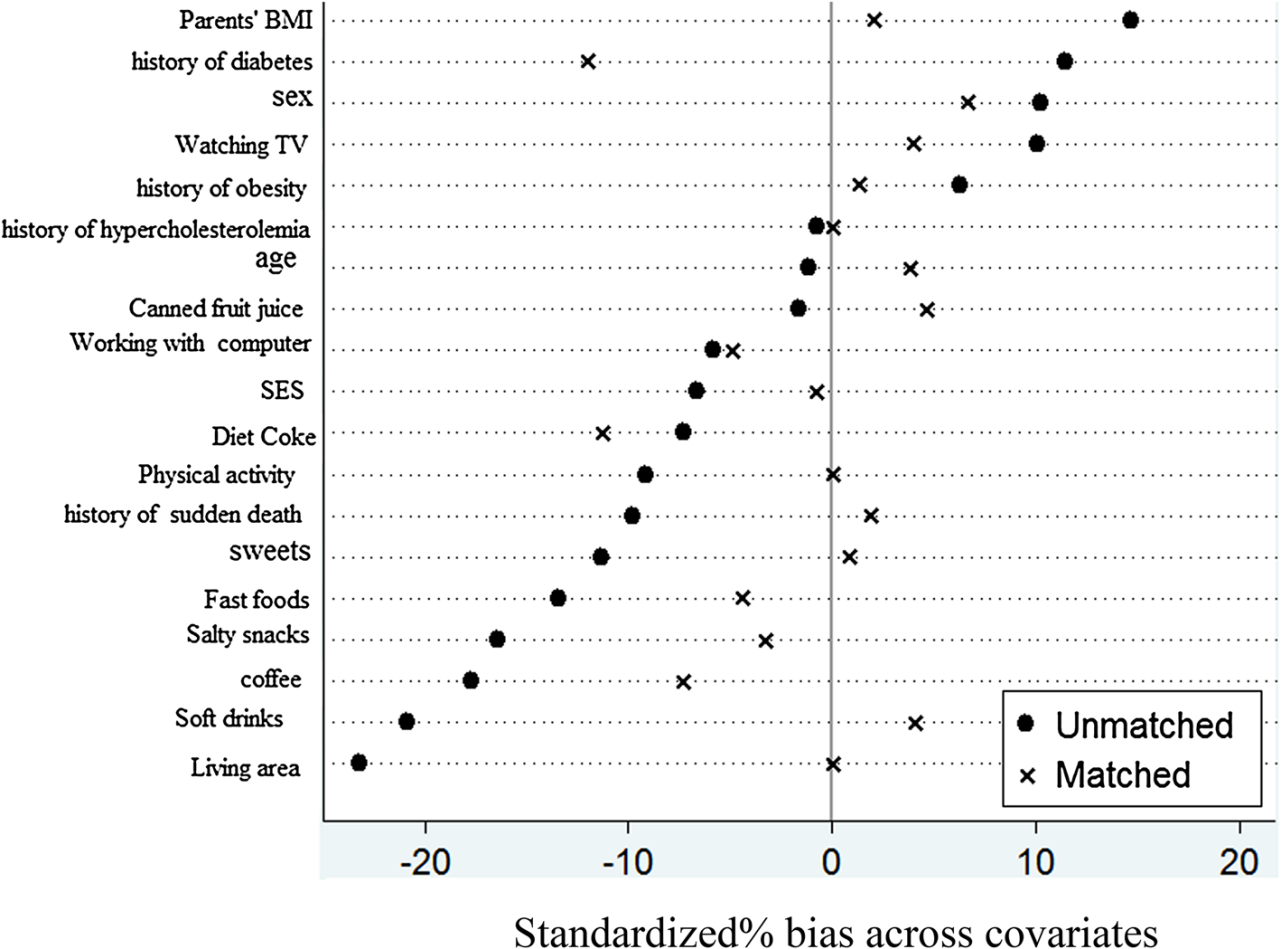
Standardized differences for general characteristics in the original and the matched sample (to assess the relationship between sleep duration and metabolic syndrome)

The PS was calculated according to logistic regression model with potential confounders. Two groups (subject with and without MetS) were matched using one to one PS matching method. Table 1 shows, the distribution of general characteristics of subjects in both groups prior to and after matching. According to PS 151 subject with Mets was matched with 151 subject without MetS. The mean of SS percentages before and after synchronization were calculated. After matching, the mean of SS percentage was decreased from 10.4 to 3.9% which shows the high quality of the matching and ensures that the distribution of general characteristics was not statistically significant between two groups (Fig. 1).

**Table 1:** General characteristics of students with and without metabolic syndrome before and after matching

Variables	Before propensity score-matched			After propensity score-matched		
	Without MetS (n = 3544)	With MetS (n = 188)	P-value	Without MetS (n = 151)	With MetS (n = 151)	P-value*
	N (%)	N (%)		N (%)	N (%)	
Age (years)						
Mean (SD)	12.44 (3.05)	12.41 (2.89)	0.88	12.26 (2.99)	12.38 (2.97)	0.74
Sex						
Boy	1855 (52.4)	108 (57.4)	0.17	85 (56.29)	90 (59.60)	0.56
Girl	1688 (47.6)	80 (42.6)		66 (43.71)	61 (40.40)	
Living area						
Urban	2560 (72.2)	154 (81.9)	0	123 (81.46)	123 (81.46)	1
Rural	984 (27.8)	34 (18.1)		28 (18.54)	28 (18.54)	
Physical activity						
Low	1116 (33.6)	60 (33.7)	0.08	59 (39.07)	51 (33.77)	0.15
Moderate	1091 (32.8)	71 (39.9)		44 (29.14)	60 (39.74)	
High	1116 (33.6)	47 (26.4)		48 (31.79)	40 (26.49)	
SES						
Low	1143 (33.8)	66 (36.7)	0.68	60 (39.74)	57 (37.75)	0.68
Moderate	1122 (33.1)	59 (32.8)		44 (29.14)	51 (33.77)	
High	1121 (33.1)	55 (30.6)		47 (31.13)	43 (28.48)	
Watching TV						
Low	1665 (47)	79 (42)	0.18	69 (45.70)	66 (43.71)	0.73
High	1875 (53)	109 (58)		82 (54.30)	85 (56.29)	
Working with computer						
Low	3161 (91.4)	172 (93)	0.46	138 (91.39)	140 (92.72)	0.67
High	297 (8.6)	13 (7)		13 (8.61)	11 (7.28)	
Parental BMI						
Mean (SD)	26.32 (4.85)	27.05 (4.99)	0.05	27.09 (4.95)	27.19 (4.88)	0.86*
Sweets						
Daily	720 (20.3)	42 (22.3)	0.19	40 (26.49)	35 (23.18)	0.78
Weekly	1531 (43.2)	91 (48.4)		66 (43.71)	75 (49.67)	
Rarely	1195 (33.7)	49 (26.1)		43 (28.48)	39 (25.83)	
Never	98 (2.8)	6 (3.2)		2 (1.32)	2 (1.32)	
Salty snacks						
Daily	162 (4.6)	19 (10.1)	0.01	11 (7.28)	13 (8.61)	0.8
Weekly	1000 (28.3)	54 (28.7)		47 (31.13)	43 (28.48)	
Rarely	1819 (51.5)	89 (47.3)		72 (47.68)	78 (51.66)	
Never	550 (15.6)	26 (13.8)		21 (13.91)	17 (11.26)	
Soft drinks						
Daily	109 (3.1)	8 (4.3)	0.01	7 (4.64)	6 (3.97)	0.91
Weekly	927 (26.2)	68 (36.2)		50 (33.11)	46 (30.46)	
Rarely	1611 (45.5)	73 (38.8)		59 (39.07)	65 (43.05)	
Never	897 (25.3)	39 (20.7)		35 (23.18)	34 (22.52)	
Diet coke						
Daily	20 (0.6)	1 (0.5)	0.76	0	1 (0.66)	0.58
Weekly	151 (4.3)	10 (5.4)		7 (4.64)	9 (5.96)	
Rarely	643 (18.2)	38 (20.4)		25 (16.56)	28 (18.54)	
Never	2710 (76.9)	137 (73.7)		119 (78.81)	113 (74.83)	
Canned fruit juice						
Daily	363 (10.4)	23 (12.4)	0.8	24 (15.89)	22 (14.57)	0.97
Weekly	1156 (33.1)	57 (30.8)		53 (35.1)	53 (35.10)	
Rarely	1501 (42.9)	79 (42.7)		62 (41.06)	62 (41.06)	
Never	476 (13.6)	26 (14.1)		12 (7.95)	14 (9.27)	
Fast foods						
Daily	418 (11.8)	28 (15)	0.35	17 (11.26)	19 (12.58)	0.98
Weekly	532 (15)	32 (17.1)		26 (17.22)	26 (17.22)	
Rarely	1898 (53.6)	97 (51.9)		82 (54.30)	82 (54.30)	
Never	696 (19.6)	30 (16)		26 (17.22)	24 (15.89)	
Coffee						
Daily	187 (5.3)	18 (9.6)	0.05	8 (5.30)	10 (6.62)	0.59
Weekly	394 (11.1)	24 (12.8)		22 (14.57)	19 (12.58)	
Rarely	1386 (39.1)	73 (38.8)		55 (36.42)	65 (43.05)	
Never	1575 (44.5)	73 (38.8)		66 (43.71)	57 (37.75)	
Family history of hyperlipidemia						
No	1719 (49.1)	92 (49.5)	0.92	77 (50.99)	77 (50.99)	1
Yes	1783 (50.9)	94 (50.5)		74 (49.01)	74 (49.01)	
Family history of diabetes						
No	1641 (46.6)	77 (41)	0.13	51 (33.77)	60 (39.74)	0.28
Yes	1877 (53.4)	111 (59)		100 (66.23)	91 (60.26)	
Family history of obesity						
No	1817 (51.5)	91 (48.4)	0.4	72 (47.68)	71 (47.02)	0.91
Yes	1708 (48.5)	97 (51.6)		79 (52.32)	80 (52.98)	

*BMI* body mass index, *SES* socioeconomic status, *MetS* metabolic syndrome

* P-vale for age according to the T-test, others are based on Chi square test

Results of multivariate conditional logistic regression for association between short sleep and MetS and its components are presented in Table 2. In the multivariate conditional logistic regression model, individuals who slept less than 8 h a day had significantly higher odds of MetS (OR 2.05, 95% CI 1.19–3.63) and high BP (OR 1.46, 95% CI 1.04–2.06). Association between short sleep duration with other MetS components (including abdominal obesity, High TG, High FBS, and low HDL-C was not statistically significant (P > 0.05). Moreover, association between the onset of sleep with MetS and its components was not statistically significant (P > 0.05).

**Table 2:** The results of the conditional logistic of sleep duration relationship with the metabolic syndrome and its components

	Dependent variable	Number of matching	Standard error of mean %	Adjusted OR (95% CI)^a^	P-value
Sleep duration (short/normal)	Metabolic Syndrome	151	3.9	*2.05 (1.19–3.63)*	*0.006*
	Low HDL-C	906	2.3	0.83 (0.68–1.02)	0.07
	High BP	298	4	*1.46 (1.04–2.06)*	*0.02*
	Abdominal obesity	624	2.1	1.16 (0.91–1.49)	0.22
	High FBG	129	7.7	1.12 (0.63–2.00)	0.68
	High TG	849	2	1.10 (0.90–1.36)	0.33
The onset of sleep (hour)	Metabolic Syndrome	153	7.2	1.29 (0.81–2.09)	0.26
	Low HDL-C	919	2	0.88 (0.73–1.07)	0.19
	High BP	297	4.5	0.97 (0.69–1.37)	0.87
	Abdominal obesity	625	3.2	1.10 (0.88–1.40)	0.4
	High FBG	131	6.5	1.40 (0.81–2.44)	0.19
	High TG	860	2.5	1.06 (0.87–1.30)	0.55

*HDL-C* high-density lipoprotein cholesterol (mg/dL), *TG* triglyceride (mg/dL), *FBG* fasting blood glucose (mg/dL), *BP* blood pressure (mmHg)

Criterion for diagnosing Components of the metabolic syndrome was obtained based on Adult Treatment Panel III: TG ≥ 110 mg/dL, HDL-C ≤ 40 mg/dL, WHR greater than 0.5, FBG ≥ 100 mg/dL, and SBP or DBP ≥ 90th percentile for age, sex and height

^a^Adjusted for age, sex, living area, SES, PA, ST, healthy and unhealthy eating behaviors, family history of chronic diseases and parental BMI

## Discussion

In the present study, we observed a significant association between short sleep duration and MetS and high BP. Individuals who slept less than 8 h a day had significantly higher Odds for MetS and high BP. Moreover, we did not find significant relationship between short sleep duration and risk of abdominal obesity, hypertriglyceridemia, hyperglycemia, and low levels of HDL-C. Also our results showed that there was no significant association between the onset of sleep with the MetS and its components.

The relation between sleep duration and MetS is controversial. Evidence regarding an association between sleep duration and the MetS in children and adolescents is sparse [38]. In accordance with our findings, a cross-sectional study reported that shorter sleep duration was associated with a higher risk of the MetS among American obese adolescents [7]. Some population-based cross-sectional studies in pediatric population have failed to find a relationship between short sleep duration with the risk of MetS [14, 39]. A dose–response meta-analysis in adult population reported that, every 1 h decrease in sleep duration corresponds to a 0.06 increase in the odds of having MetS [40]. A cohort study of 3974 Brazilian adolescents aged 11 years found that sleep deprivation may have adverse metabolic effects later in life [41].

Several mechanisms have been reported to explain the relationship of short sleep duration with obesity. Sleep deprivation may be associated with upregulation of energy-expenditure reduction, appetite, increasing time spent eating and adiposity. This association may be mediated by increasing levels of ghrelin, decreasing levels of leptin, an increased ratio of ghrelin to leptin, low levels of testosterone, and high levels of cortisol. Additionally, dysregulation of the autonomic nervous system may play a role in the association between sleep deprivation and obesity, which is mediated by elevation of hypothalamic-pituitary-adrenal axis activity and activated systemic inflammatory processes [17, 42].

In our study we did not find significant relationship between short sleep duration and risk of abdominal obesity, hypertriglyceridemia, hyperglycemia, and low levels of HDL-C.

The results of a cross-sectional study among American obese children and adolescents have reported that sleep duration does not predict the development of MetS, adiposity, and insulin resistance [14]. Another cohort study in Brazilian adolescents have shown, reduction in time in bed was not associated with changes in WC over a 1 year period in 6th grade adolescents [43]. However previous prospective cohort studies reported that short sleep duration is associated with the risk of central obesity in children [18, 44]. The majority of cross-sectional studies have indicated that short sleep duration (generally < 5 h per night) enhances the risk for abdominal obesity in both adults and children in different countries [17, 39].

There was no association between sleep duration and high FBS and high TG in the current study. A clinical based cross-sectional study in American adolescents revealed that sleep durations was not associated with high FBS but it was associated with high TG [14]. A study in Korean adolescents reported adolescents with long sleep duration showed elevated fasting glucose level, but the odds for hyperglycemia was not associated with sleep duration [39]. The mechanism underlying this relation is not clear. This can be explained by more chronic sequelae of sleep restriction may require time to develop and become manifest [38]. The opposing direction of the relations between individual components of MetS and sleep duration might be low prevalence (38.7%) of MetS components within the study population.

HDL-C did not show any relation to sleep duration. While our finding between short sleep duration and HDL-C was in the unexpected direction, interestingly, similar trends have been reported in other pediatric studies [14, 45]. However, although there is no clear mechanism as to why shorter sleep duration is associated with higher HDL-C, it is possible that sleep quality may play a role. Disruptions in circadian rhythms and subsequent poor quality sleep may contribute to dyslipidemia [46].

Among the components of MetS, we found that individuals who slept less than 8 h a day were 1.4 times more likely to have high BP. In a cross-sectional study on 238 American adolescents age 13–16 years, the odds of prehypertension was increased 2.5-fold for short sleep (< 6.5 h) adjusted for gender, BMI percentile, and socioeconomic status [47]. Another prospective study among Netherland adolescents show that sleep duration was associated with not associated with hypertension [45]. The mechanism underlying the association between sleep duration and hypertension is not fully understood. A link between insufficient sleep and metabolic changes, such as reduced glucose tolerance, have been considered to play important roles in the complicated mechanisms leading to hypertension [16]. short sleep duration could also increase the risk of hypertension indirectly through an unhealthy lifestyle [48].

To the best of our knowledge, this is the first study that evaluate association of sleep duration with MetS and its component using a robust statistical method (propensity score matching) which can control a large number of potential confounders. Our study has several limitations. First, as it was a cross-sectional study, we cannot prove any cause-effect association between sleep duration and MetS. Future cohort studies using a prospective approach is needed to determine sleep duration as a cause of MetS. Second, sleep duration was assessed using self-reported measurement instead of objective measurement which may cause misclassification of sleep duration. However, a previous study revealed that self-reported sleep duration is reasonably valid when compared to objective measurement [49]. Third, we also did not measure the quality of sleeping which is associated with MetS according to the previous studies [38, 45, 47]. Therefore, future studies, which investigate simultaneously sleep duration and sleep quality may be helpful.

## Conclusion

Our findings showed that short sleep duration is associated with increased risk of MetS and high BP in children and adolescents. Future longitudinal studies is needed to determine the clinical impact of current findings.
